# Impact of community-based interventions on HIV knowledge, attitudes, and transmission

**DOI:** 10.1186/2049-9957-3-26

**Published:** 2014-08-01

**Authors:** Rehana A Salam, Sarah Haroon, Hashim H Ahmed, Jai K Das, Zulfiqar A Bhutta

**Affiliations:** 1 Division of Women and Child Health, Aga Khan University, Karachi, Pakistan; 2 Center of Excellence in Women & Child Health, The Aga Khan University, Karachi, Pakistan; 3 Center for Global Child Health Hospital for Sick Children, Toronto, Canada

**Keywords:** Community-based interventions, Antiretroviral therapy, HIV prevention, HIV/AIDS

## Abstract

In 2012, an estimated 35.3 million people lived with HIV, while approximately two million new HIV infections were reported. Community-based interventions (CBIs) for the prevention and control of HIV allow increased access and ease availability of medical care to population at risk, or already infected with, HIV. This paper evaluates the impact of CBIs on HIV knowledge, attitudes, and transmission. We included 39 studies on educational activities, counseling sessions, home visits, mentoring, women’s groups, peer leadership, and street outreach activities in community settings that aimed to increase awareness on HIV/AIDS risk factors and ensure treatment adherence. Our review findings suggest that CBIs to increase HIV awareness and risk reduction are effective in improving knowledge, attitudes, and practice outcomes as evidenced by the increased knowledge scores for HIV/AIDS (SMD: 0.66, 95% CI: 0.25, 1.07), protected sexual encounters (RR: 1.19, 95% CI: 1.13, 1.25), condom use (SMD: 0.96, 95% CI: 0.03, 1.58), and decreased frequency of sexual intercourse (RR: 0.76, 95% CI: 0.61, 0.96). Analysis shows that CBIs did not have any significant impact on scores for self-efficacy and communication. We found very limited evidence on community-based management for HIV infected population and prevention of mother- to-child transmission (MTCT) for HIV-infected pregnant women. Qualitative synthesis suggests that establishment of community support at the onset of HIV prevention programs leads to community acceptance and engagement. School-based delivery of HIV prevention education and contraceptive distribution have also been advocated as potential strategies to target high-risk youth group. Future studies should focus on evaluating the effectiveness of community delivery platforms for prevention of MTCT, and various emerging models of care to improve morbidity and mortality outcomes.

## Multilingual abstracts

Please see Additional file 1 for translations of the abstract into the six official working languages of the United Nations.

## Introduction

In 2012, an estimated 35.3 million people lived with HIV, while approximately two million new HIV infections were reported globally; a 33% decline in the number of new infections as compared to 2001 [1]. Concurrently, the number of AIDS deaths also declined from 2.3 million in 2005 to 1.6 million in 2012 [1]. As many as eight million people in low- middle- income countries (LMICs) are currently receiving lifesaving treatment [2]. In Sub-Saharan Africa, interventions to prevent HIV led to a decline in the number of newly infected children by 24% between 2009 and 2011 [3], owing to the rapid increase in access to preventive and therapeutic services for women with HIV. Notwithstanding the progress made on many fronts since the emergence of AIDS in 1981, a lot more still needs to be done. The number of new HIV infections among children was 210,000; five out of 10 women or their infants did not receive antiretroviral (ARV) medicines during breastfeeding to prevent mother-to-child transmission (MTCT); and four out of 10 pregnant women living with HIV did not receive ARV medicines to prevent MTCT, in 2012 [4]. The intricate link between tuberculosis (TB) and HIV also poses a major threat to the efforts to control both infections as people living with HIV have a 12–20 times higher risk of developing TB. The details on HIV epidemiology, burden, and transmission has been documented in our previous publication [5].

Effective HIV prevention measures should ideally emphasize human dignity, responsibility, voluntary participation, and empowerment through access to information, services and support systems [6]. A thorough understanding of common values and belief systems also helps to identify positive values and practices that can facilitate and more effectively promote HIV interventions. Hence community-based approaches are increasingly being advocated for HIV prevention. Community-based interventions (CBIs) are built on shared values and norms, and belief systems and social practices, and permit culturally sensitive discussions of HIV, and sexual and reproductive health. They allow increased access and ease availability of medical care to population potentially at risk of, or already infected with HIV by reaching individuals in homes, schools, or community centres. CBIs involve education and counseling to promote HIV awareness and risk-reducing behaviors, promotion of HIV testing and counseling, administering of appropriate treatment to HIV-infected mothers to prevent MTCT, micronutrient supplementation for pregnant and lactating women and interventions to increase adherence to treatment via home visits. Nonetheless, the nature and scale of CBIs vary according to the type of the HIV epidemic scenario. In hyperendemic situations and generalized epidemics, extraordinary efforts are required to mobilize the whole community. In low-prevalence countries and concentrated epidemics, CBIs should focus on reaching those groups that are most at risk [6].

This paper aims to systematically analyze the effectiveness of CBIs for the prevention and management of HIV, including education and counseling, adherence to treatment and MTCT.

## Methods

We systematically reviewed literature published before July 2013 to identify randomized controlled trials (RCTs), quasi-experimental, and before-and-after studies on CBIs for the prevention and management of HIV. Studies were included if intervention was delivered within community settings and reported outcomes were relevant to the review. We excluded studies if any component of the intervention was delivered at a health facility; if the interventions targeted special populations including sex workers, men who have sex with men, injection drug users, prisoners, bar workers, patients with mental illness and armed forces; or if the objective was to evaluate process outcomes. Search was conducted in PubMed, Cochrane libraries, Embase, and the World Health Organization (WHO) Regional Databases to identify all published and unpublished studies. Additional studies were identified by manually searching references from the included studies. Studies that met the inclusion criteria were selected and double data abstracted on a standardized abstraction sheet. Quality assessment of the included RCTs was done using the Cochrane risk of bias assessment tool [7]. We conducted meta-analysis for individual studies using the software Review Manager 5.1. Pooled statistics were reported as relative risk (RR) for categorical variables and standard mean difference (SMD) for continuous variables between the experimental and control groups with 95% confidence intervals (CIs). The outcomes of interest included knowledge, attitudes, and behavior outcomes; birth outcomes; HIV transmission; and morbidity and mortality. These are outlined in Table 1. We also attempted to qualitatively synthesize the findings reported in the included studies for other pragmatic parameters identified in our conceptual framework including intervention coverage, challenges/barriers, enabling factors, aspects related to the integrated delivery, monitoring and evaluation and equity. The detailed methodology is described in Paper 2 of this series [8].

**Table 1:** Outcomes analyzed

***Outcomes***	***Outcomes analyzed***
**Knowledge, attitudes, and behaviors**	Knowledge about HIV/AIDS and risk reduction
	Self-efficacy
	Communication
	Engaging in sexual intercourse
	Protected sex
	Treatment adherence
**Birth outcomes**	Low birth weight
	Stillbirth
**HIV transmission**	HIV infection at birth
	HIV infection among infants with/without breastfeeding
**Morbidity and mortality**	Detectable viral load
	All-cause mortality
	Cause-specific mortality

## Review

**Figure 1:**
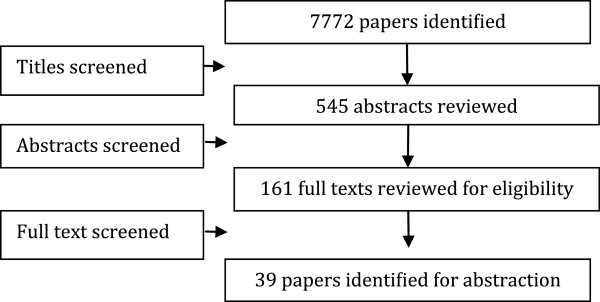
Search flow diagram.

We identified 7,772 titles from the search conducted in all databases. After screening titles and abstracts, 161 full texts were reviewed; of which 39 studies (Figure 1) [9-35] were selected for inclusion. These included 18 RCTs, 14 quasi-experimental studies, and seven before-and-after studies. Nine studies could not be included in the meta-analysis as they did not report poolable data. For the 18 RCTs, randomization was adequate in all the studies except for one, allocation concealment and participant blinding could not be done in majority of the studies due to the nature of interventions, adequate sequence generation was not done or unclear in most of the studies, and selective reporting was not apparent in any of the studies (see Table 2).

**Table 2:** Quality assessment of the included RCTs

***Article***	***Randomization***	***Sequence generation***	***Allocation concealment***	***Blinding of participants***	***Blinding of assessors***	***Selective reporting***
Berrien 2004	Done	Done	Done	No	No	No
Carlson 2012	Done	Done	Not clear	No	No	No
Chhabra 2010	Done	Coin toss	No	No	No	No
Chen 2011	Done	Not clear	No	No	Not clear	No
Clark 2005	Not clear	No	No	No	No	No
Fawole 1999	Done	Not clear	Not clear	No	No	No
Fitzgerald 1999	Done	Not clear	Not clear	No	No	No
Huba 1999	Done	Not clear	No	No	Not clear	No
Kiene 2006	Done	Done	No	No	Not clear	No
Klepp 1997	Done	Not clear	No	No	Not clear	No
Jemmott 2010	Done	Done	No	No	Yes	No
Rotheram-Borus 1998	Done	Not Clear	No	No	Yes	No
Shapiro 2010	Done	Done	Not clear	Not clear	Not clear	No
Selke 2010	Done	Not clear	No	No	Not clear	No
Walker 2004	Done	Done	Not clear	No	Not clear	No
Williams 2006	Done	Done	No	No	Done	No

Included studies mainly focused on community-based HIV prevention through educational activities, counseling sessions, home visits, mentoring, women’s groups, peer-leadership, custom computerized HIV/AIDS risk reduction, and street outreach activities and address perceived barriers to counseling and voluntary testing. Among studies conducted on known HIV cases, three studies provided home healthcare to HIV-infected adults to improve general health and treatment adherence, one study evaluated the impact of community delivered ARV regimens during pregnancy and breast feeding, and one study utilized computer-based technologies including Personal Digital Assistant to support home assessments for HIV-infected adults. Most of the studies targeted adolescents and youth, while some targeted HIV-infected population in general, urban working women, and high-risk heterosexual men. All of the studies were non-integrated including six studies that were school-based. The characteristics of the included studies are summarized in Table 3.

**Table 3:** Characteristics of the included studies

***Study***	***Study design***	***Country***	***Intervention***	***Target Population***	***Integrated/non-Integrated***
Agarwal 2004	Pre-post study	India	Health education about the prevention of reproductive tract infection and HIV/AIDS imparted through one-to-one interactions with men and women during home visits, at village-based clinics and health camps, and through health-education talks with men and women	Men and women of reproductive age	Non-integrated
Baptiste 2006	Quasi- experimental	South Africa, Trinidad, and Tobago	Community participatory, family-based prevention (CHAMP)	Youth	Non-integrated
Berrien 2004	RCT	USA	Eight structured home visits for education and counseling to improve adherence over a three-month period by a registered nurse	HIV-positive children and youth (aged 7 years and above)	Non-integrated
Blake 2003	Pre-post	USA	Condom availability in high schools, and community discussion and involvement for HIV prevention	Adolescents	Non-integrated
Carlson 2012	cRCT	Tanzania	28-week course in health curriculum, two-to-three hour weekly sessions on HIV/AIDS competence and other subjects (citizenship, community health, social ecology)	Adolescents aged 9–14 years	School-based non-integrated
Chen 2011	RCT	Bahamas	Teaching sessions involving parents	Youth	School-based non-integrated
Chhabra 2010	RCT	India	HIV/AIDS and alcohol abuse educational program designed keeping in mind cultural, linguistic, and community-specific characteristics. A single one-hour session per week for 10 consecutive weeks.	Rural and tribal youth aged 13–16 years, in schools	School-based non-integrated
Clark 2005	RCT	USA	Ten sessions on adult identity mentoring, conducted once or twice a week for six weeks	African-American seventh-grader students	School based non-integrated
Fawole 1999	RCT	Nigeria	Health education initiatives to increase HIV knowledge and sexual practices	School children	School-based non-integrated
Fitzgerald 1999	RCT	Namibia	14-sessions of face-to face intervention emphasizing abstinence and safer sex for HIV prevention	Youth	Non-integrated
Harper 2009	Quasi- experimental	USA	Nine sessions of community-based, culturally- and ecologically-tailored HIV prevention intervention (SHERO)	Mexican-American female adolescents aged 12–21 years	Non-integrated
Heitgerd 2011	Pre-post study	USA	Community-based small group discussions on healthy relations	People living with HIV	Non-integrated
Huba 1999	RCT	USA	Home healthcare via home visits by multi-disciplinary teams	People living with HIV	Non-integrated
Jemmott 2010	cRCT	South Africa	Two six-session interventions based on behavior-change theories on HIV/STD risk-reduction targeted at sexual risk behaviors	Sixth-grade students	School-based non-integrate
Jemmott 1998	RCT	USA	Abstinence and safe sex HIV risk reduction intervention	African-American adolescents	Non-integrated
Kiene 2006	RCT	US	A custom computerized HIV/AIDS risk reduction intervention to increase HIV/AIDS preventive behaviors	General population	Non-integrated
Kinsler 2004	Quasi- experimental	Belize	Cognitive-behavioral peer-facilitated school-based HIV/AIDS education program	School children	Non-integrated school- based
Kinsman 2001	Quasi- experimental	Uganda	Non-integrated school-based program	School children	Non-integrated school- based
Klepp 1997	RCT	Tanzania	Program to reduce children’s risk of HIV infection, and improve tolerance and care towards HIV patients	Sixth-grade students	Non-integrated school- based
Li 2012	Quasi- experimental	China	School-based curriculum for HIV prevention education	School children	Non-integrated school- based
Maticka-Tyndale 2007	Quasi- experimental	Kenya	Primary-school HIV education initiative on the knowledge, self-efficacy and sexual practices, and condom use	School children	Non-integrated school- based
Mcbride 2007	Quasi- experimental	USA	Family-based HIV preventive intervention (CHAMP)	African-American youth	Non-integrated
Merakou 2006	Quasi- experimental	Greece	Peer-education intervention	Adolescents	Non-integrated school- based
Middelkoop 2006	Quasi- experimental	South Africa	Young adults from the community received training in HIV/AIDS and drama, and developed sketches to address perceived barriers to voluntary counseling and testing	Young adults and community members	Non-integrated
Morisky 2004	Quasi- experimental	Philippines	Participatory action research to change high-risk sexual behaviors	Heterosexual men	Non-integrated
Munodawafa 1995	Quasi- experimental	Zimbabwe	Health instruction provided by student nurses on prevention of STDs, HIV/AIDS, and drugs	School children	Non-integrated school- based
Murdock 2003	Pre-post study	South Africa	Female-led HIV workshops	Women	Non-integrated
Nelson 2012	Pre-post study	USA	Native Voice Intervention: four-day workshop on substance abuse, HIV, and hepatitis prevention	American Indian/Alaska native youth	Non-integrated
Norr 2004	Quasi-experimental	Botswana	Peer-group HIV prevention intervention based on social–cognitive learning theory, gender inequality, and the primary health care model for community-based health promotion	Urban employed women	Non-integrated
Okonofua 2003	RCT	Nigeria	Community participation, peer education, public lectures, health clubs in the schools, and training of STD treatment providers	Adolescents	Non-integrated
Pearlman 2002	Quasi- experimental	USA	Community-based HIV/AIDS peer leadership prevention program	Adolescents	Non-integrated
Rotheram-Borus 1998	RCT	USA	Education sessions: a seven-session intervention of 1.5 hours each or a three-session intervention of 3.5 hours each	Adolescent aged 13–24 years	Non-integrated
Selke 2010	cRCT	Kenya	The intervention group received monthly Personal Digital Assistant for supported home assessments	Adult with HIV on ART	Non-integrated
Shapiro 2010	RCT	Southern Botswana	300 mg of Abacavir, 300 mg of Zidovudine, and 150 mg of Lamivudine twice daily (the NRTI group), or 400 mg of Lopinavir and 100 mg of Ritonavir co-formulated as Kaletra (Abbott) with 300 mg of Zidovudine and 150 mg of Lamivudine twice daily (the protease-inhibitor group) from 26 to 34 weeks’ gestation through planned weaning by six months post partum	HIV-infected women between 26 and 34 weeks’ gestation	Non-integrated
Villarruel 2006	Pre-post study	Philadelphia, USA	HIV and health-promotion control interventions consisting of six 50-minute modules delivered by adult facilitators to small, mixed-gender groups	Adolescents	Non-integrated
Visser 2005	Pre-post study	South Africa	Life skills training and HIV/AIDS education in schools as part of the school curriculum	Adolescents	School-based non-integrated
Walker 2004	RCT	Mexico	HIV prevention course that promoted condom use, the same course with emergency contraception as back-up, or the existing sex education course	Adolescents	School-based non-integrated
Williams 2006	RCT	USA	Community-based, home-visit intervention to improve medication adherence	Adults with HIV on ART	Non-integrated
Wendell 2003	Quasi-experimental	USA	Street outreach intervention to improve risk behaviors	General population	Non-integrated

### Quantitative synthesis

**Figure 2:**
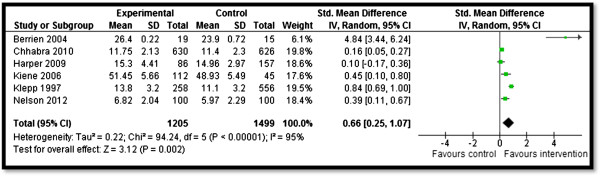
Forest plot for the impact of CBIs on knowledge.

**Figure 3:**
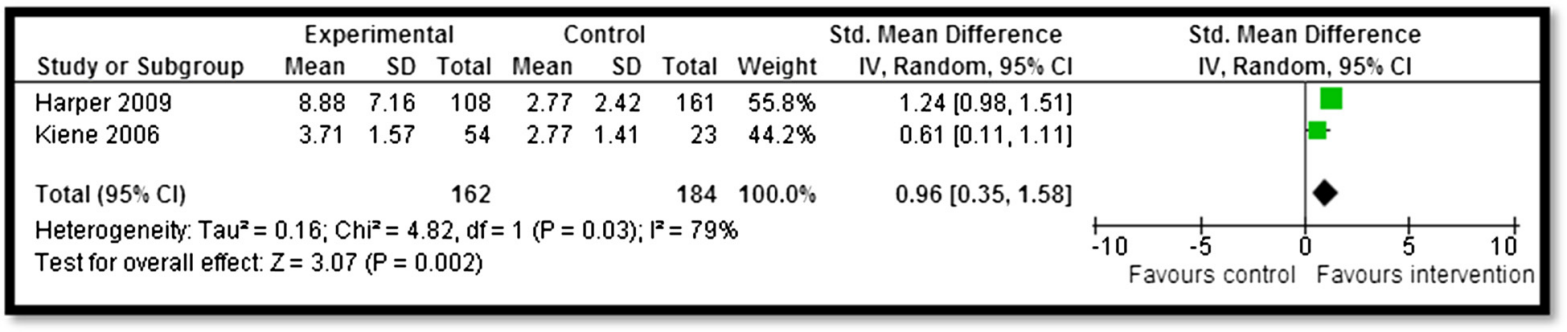
Forest plot for the impact of CBIs on condom use.

**Figure 4:**
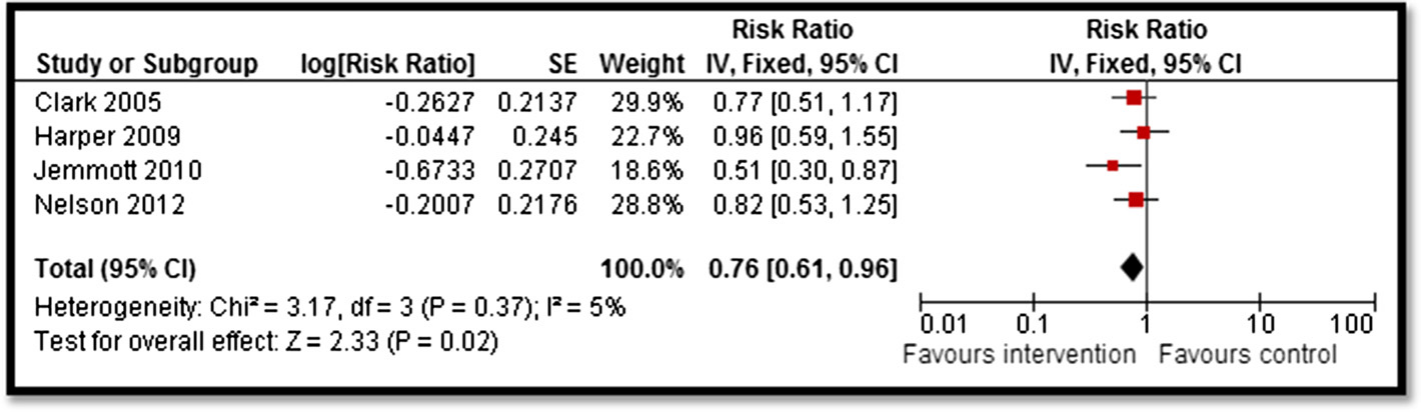
Forest plot for the impact of CBIs on sexual activity.

**Figure 5:**
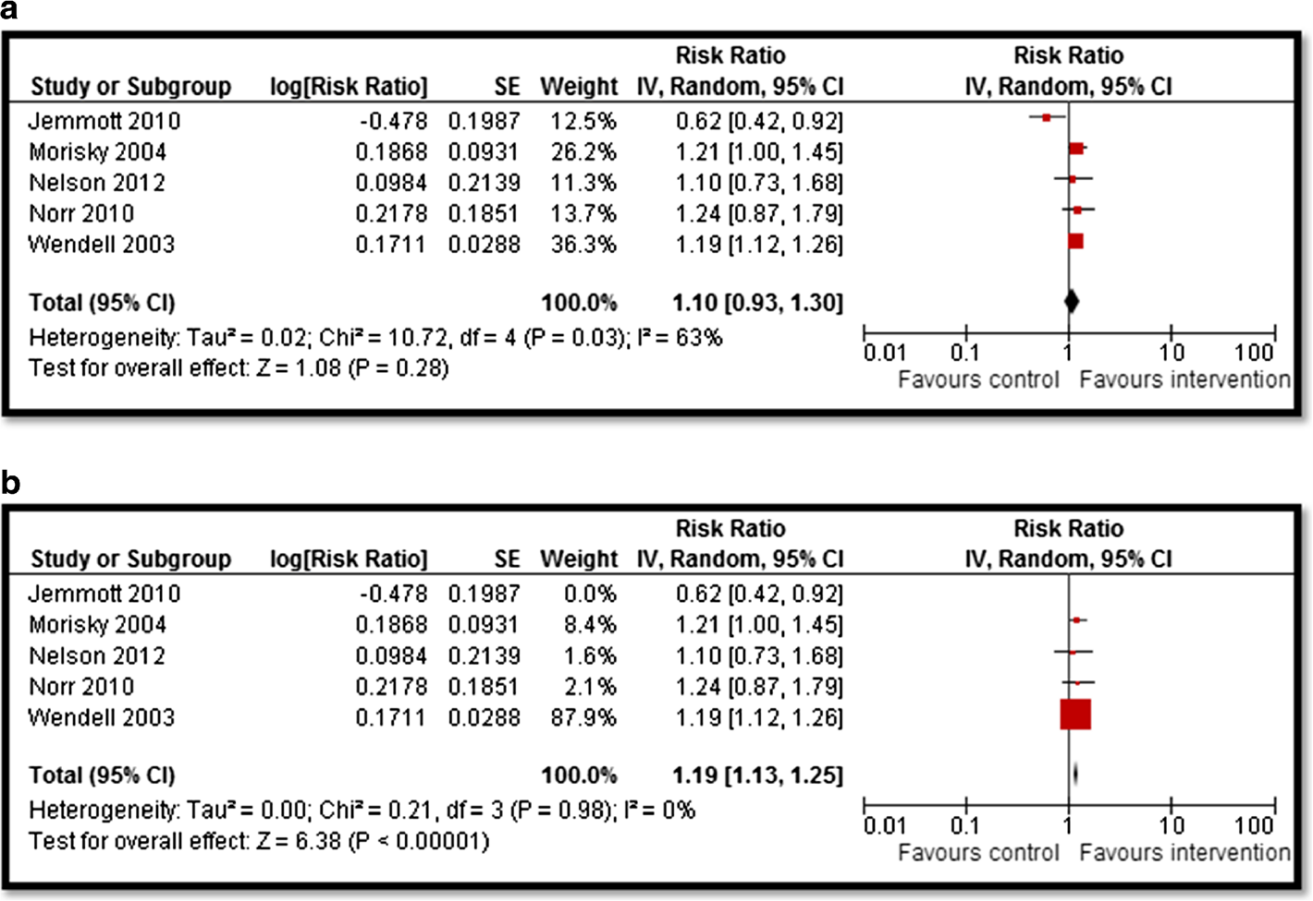
Forest plot for the impact of CBIs on protected sex (a) with all studies, (b) after sensitivity analysis.

Table 4 summarizes the quantitative findings. CBIs to increase awareness about HIV/AIDS risk factors and promote preventive measures resulted in significant improvement in outcomes related to HIV/AIDS knowledge, attitudes, and behavior. Community delivered interventions such as HIV/AIDS education and counseling during home visits, educational programs built on community specific characteristics, and computer-based HIV risk reduction interventions significantly improved participants’ knowledge scores (SMD: 0.66; 95% CI: 0.25, 1.07) for HIV/AIDS (see Figure 2). Community-based culturally and ecologically tailored HIV prevention interventions and custom computerized HIV risk reduction interventions resulted in a significantly increased condom use (SMD: 0.96; 95% CI: 0.03, 1.58) among the target population (see Figure 3). Community education on abstinence and safe sex, and adult identity mentoring for preventing HIV risk behaviors led to a significant decrease in sexual activity (RR: 0.76; 95% CI: 0.61, 0.96) (see Figure 4). The frequency of protected sex increased by 19% (RR: 1.19; 95% CI: 1.13, 1.25), with street outreach activities and peer-group education on abstinence and HIV risk reduction. However, this finding was reported in sensitivity analysis conducted after removing Jemmott 2010 [28] due to high heterogeneity, and because this study proved to be an outlier on visual inspection (see Figure 5a and 5b). Our analysis did not find any impact of CBIs on scores for self-efficacy, risk taking, and communication.

**Table 4:** Summary estimates for the overall and subgroup analysis for school-based, non-integrated, and integrated delivery strategies

**Outcomes**	**Estimates**
**Knowledge, attitudes, and practices**	
HIV/AIDS related knowledge	**0.66 [0.25, 1.07]** 6 datasets, 6 studies
Self-efficacy	0.42 [-0.09, 0.93] 4 datasets, 4 studies
Communication	-0.10 [-0.56, 0.35] 5 datasets, 5 studies
Risk taking	-0.18 [-0.43, 0.07] 1 dataset, 1 study
Engaging in sexual intercourse	**0.76 [0.61, 0.96]** 4 datasets, 4 studies
Protected sex	1.10 [0.93, 1.30] 5 datasets, 5 studies
Protected sex (with sensitivity analysis)	**1.19 [1.13, 1.25]** 4 datasets, 4 studies
Mean number of times condoms used	**0.96 [0.35, 1.58]** 2 datasets, 2 studies
Treatment adherence score	**3.88 [2.69, 5.07]** 1 dataset, 1 study
**Birth outcomes**	
Low birth weight	0.92 [0.68, 1.24] 2 datasets, 1 study
Stillbirth	**0.34 [0.18, 0.65]** 2 datasets, 1 study
**HIV transmission**	
HIV at birth	1.32 [0.24, 7.31] 2 datasets, 1 study
HIV infection at six months among breastfed infants	1.74 [0.33, 9.31] 2 datasets, 1 study
**Morbidity and Mortality**	
Detectable viral load	1.16 [0.48, 2.80] 2 datasets, 2 studies
All-cause infant mortality (in six months)	0.56 [0.27, 1.17] 2 datasets, 1 study

*estimates in bold are statistically significant.

**Figure 6:**
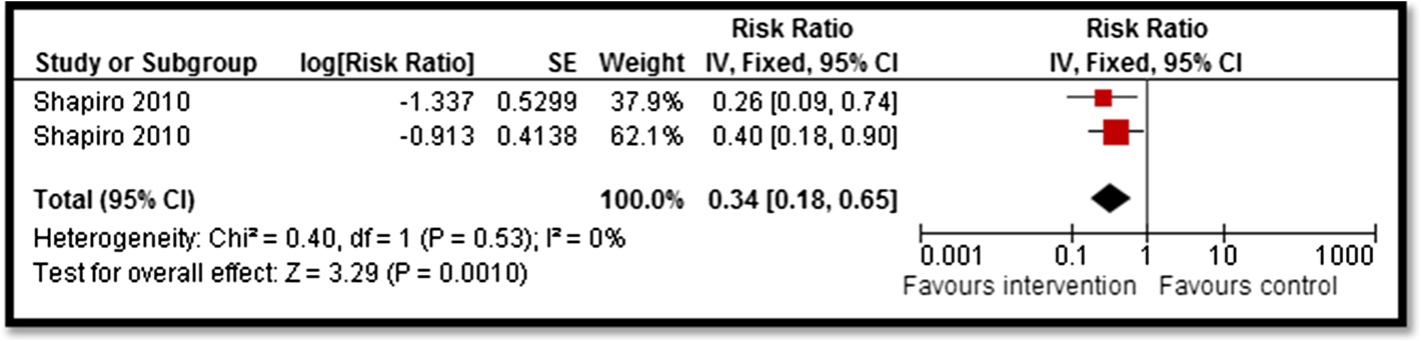
Forest plot for the impact of CBIs on stillbirth.

We found limited evidence on the effectiveness of CBIs for the management of HIV-infected population. Home visits for HIV patients to improve treatment adherence and general health outcomes led to a significant increase in treatment adherence score (MD: 3.88; 95% CI: 2.69–5.07), however, this finding is based on a single study. One study evaluated community-based delivery of highly- active antiretroviral therapy (HAART) during pregnancy and lactation to prevent MTCT [33]. It reported significant decrease in stillbirths by 66% (RR 0.34; 95% CI: 0.18, 0.65) (see Figure 6), while there were no significant impacts on low birth weight (LBW), and HIV transmission at birth or at six months among breastfeeding infants. We did not find any impact of CBIs on morbidity and mortality outcomes.

### Qualitative synthesis

Community support and mobilization have been reported as the key enabling factors for the success of CBIs for HIV prevention since they require a culturally sensitive approach [24,25]. Localized intervention strategies aimed at community mobilization were found to be effective and sustainable when delivered within the context of an existing or emergent public health system and linked to other programs in the community [25]. Most of the studies focusing on the prevention of HIV risk behaviors targeted adolescents and highlighted the significance of culturally grounded HIV prevention programs created in collaboration with community members to address adolescent sexual behaviors and prevent unhealthy sexual practices [13]. Culturally sensitive educational interventions have reported increased knowledge, efficacy, confidence and communication skills, and decreased risky behaviors [25]. The establishment of community support at the onset of such programs led to acceptance and engagement of the community in HIV prevention efforts even in remote and less industrialized areas [25,36]. Emphasizing citizenship skills, active participation, and decision making promoted adolescent participation in HIV prevention programs targeting young people [24]. Continuous involvement from the former participants and facilitators in education and community development are also key components to increase coverage and participation [24]. School-based delivery of HIV prevention education and contraceptive distribution have also been advocated as strategies to target the high-risk youth group. Studies support using teachers as life skills presenters because they have contact with the students on an ongoing basis, which contributes to the sustainability of the program [36]. However, teachers require a lot of support from the project teams to facilitate change.

Included studies suggest that home-based interventions can achieve better adherence to prescribed medication regimens among HIV-positive children, adults, and their families as it allows their patients and caretakers to better understand HIV infection and ARV medications [11,23]. Home-based delivery of ART and health education by nurses help to build trusting and accepting relationships between nurses and families, which can ensure successful adherence [11].

One of the major barriers in implementing programs for HIV prevention and screening is the traditional cultural beliefs and reluctance to talk about sexual issues. This poses a major roadblock in the development of HIV education programs [25]. These barriers could be addressed if the community was involved from the very inception of such programs and provided an opportunity to design initiatives that are sensitive to their culture and beliefs. Schools-based HIV prevention programs are also faced with issues such as maintaining specific standards of safety, discipline and educational attainment, and often lack resources for HIV prevention interventions. Low teacher involvement, lack of human resources, and low awareness and commitment to deal with the problem makes school-based delivery difficult [36]. When designing school-based programs, regional differences need to be taken into account as some schools might be more comfortable with a single sex delivery for HIV prevention interventions [25]. Furthermore, despite the intensive training, teachers rarely change their preconceptions about adolescent sexuality [37]. Compounding these problems is the issue that many adolescents lack strong role models and mentors to guide them through the exploration that naturally occurs as a part of adolescent self-identity development, thus potentially leading to unhealthy and risky sexual practices [26].

## Discussion

Our review findings suggest that CBIs to increase HIV awareness and risk reduction interventions are effective in improving knowledge, attitudes, and practice outcomes as evidenced by increased knowledge scores for HIV/AIDS, protected sexual encounters, condom use, and decreased frequency of sexual intercourse. CBIs did not show any impact on scores for self-efficacy and communication. We found very limited evidence on community-based management programs for HIV-infected population and prevention of MTCT for HIV-infected pregnant women. Existing evidence from a single study suggests that healthcare and treatment via home visitations have the potential to improve adherence to ART regimen. Community-based provision of HAART to HIV-positive pregnant women led to significant decrease in stillbirths, although these findings are based on a single study. We did not find any impact of CBIs on prevention of MTCT, LBW, and HIV/AIDS associated morbidity and mortality. We could not conduct any subgroup analyses for the relative effectiveness of integrated and non-integrated delivery strategies in our review since all the studies were delivered in non-integrated manner. Existing systematic reviews on community-based HIV/AIDS prevention and control programs are limited in their scope as they either evaluate the effectiveness of a single intervention, or interventions targeted at a specific population group [38-43].

With HIV still being a global epidemic, it is crucial that efforts be undertaken to utilize existing community-based infrastructure to introduce interventions for HIV prevention and also target the most vulnerable population groups. Many of the risk factors for HIV/AIDS including drug abuse and unsafe sexual practices are initiated in the adolescent age group. Targeting preventive interventions towards adolescence presents a window of opportunity to reduce the future burden of HIV/AIDS and allows time for maximum impact on health to be achieved in the years ahead. Based on our review findings, community-based preventive health education and counseling, abstinence and HIV risk reduction, and street outreach interventions are effective in improving a range of knowledge, attitudes, and behavior outcomes. These interventions should be scaled-up at the community level to target high-risk population groups, including adolescents, to improve HIV/AIDS related knowledge and modify sexual risk behaviors to prevent HIV. However, implementation, scaling-up, and sustainability may be difficult to achieve and need careful consideration [44-47].

We found a dearth of evidence on the effectiveness of CBIs targeting HIV-infected population groups, and pregnant and lactating women living with HIV. Targeting pregnant women with HIV is critical as prevention of MTCT would not be possible if this group remains neglected [48]. The coverage of effective ART regimens in LMICs for preventing MTCT was 57% in 2011, and a lot still needs to be done to eliminate it completely. Nearly half of all children newly infected with HIV in 20 countries in Africa are acquiring HIV during breastfeeding because of the low ART coverage their mothers receive. Various community delivery models for targeting pregnant women with HIV need to be evaluated for effectiveness to improve birth outcomes, HIV transmission, and maternal and neonatal morbidity and mortality. Integrating HIV voluntary testing, counseling, and treatment with routine community delivered antenatal care (ANC) in high-risk areas could potentially improve coverage and reduce the risk of MTCT during pregnancy and lactation. In the 21 priority countries in Sub-Saharan Africa, services to prevent new HIV infections among children have been integrated into existing maternal and child health care [49]. Greater attention should be paid to the period before pregnancy to improve the rates of voluntary testing and counselling to prevent MTCT [50].

With the increasing risk of TB resurgence associated with HIV, various HIV and TB integrated models have also been proposed. The WHO estimates that the scale-up of collaborative HIV/TB activities (including HIV testing, ART, and recommended preventive measures) have stopped 1.3 million people from dying from 2005 to 2012 [1]. However, challenges persist, as progress in reducing TB-related deaths among people living with HIV has slowed in recent years [1]. In 2012, South Africa launched an integrated five-year strategy addressing HIV, TB, and sexually- transmitted infections. Similarly, in Malawi, the number of facilities providing integrated HIV and sexual and reproductive health services have increased [49]. Large-scale community intervention models have been launched in Malawi, Mozambique, and South Africa involving decentralization of care and delegation to non-clinician physicians [51]. However, there is still a need to rigorously evaluate the emerging new models of care for effectiveness in order to improve morbidity and mortality outcomes.

## Conclusion

CBIs are effective in improving knowledge, attitudes, and practice outcomes. Future studies should focus on evaluating the effectiveness of community delivery platforms for prevention of MTCT, and various emerging models of care to improve morbidity and mortality outcomes.

## Abbreviations

ANC: Antenatal care; ARV: Antiretroviral; ART: Antiretroviral therapy; CBI: Community-Based Intervention; IDoP: Infectious diseases of poverty; LMIC: Low- middle-income country; MTCT: Mother-to-Child Transmission; WHO: World Health Organization.

## Competing interests

The authors declare that they have no financial or non-financial competing interests.

## Authors’ contributions

ZAB was responsible for designing and coordinating the review. SH and HHA were responsible for the data collection, screening of the search results, screening of the retrieved papers against inclusion criteria, appraising the quality of papers, and abstracting the data. RAS and JKD were responsible for data interpretation and writing the review. ZAB critically reviewed and modified the manuscript. All authors read and approved the final manuscript.

## Supplementary Material

Additional file 1 Multilingual abstracts in the six official working languages of the United Nations. Click here for file
