# Supplementary material for: Impact of community-based interventions on HIV knowledge, attitudes, and transmission
Source: Infect Dis Poverty. 2014 Aug 1;3:26. doi: 10.1186/2049-9957-3-26 (PMC4132935; doi:10.1186/2049-9957-3-26)
Supplement: Additional file 1 — Multilingual abstracts in the six official working languages of the United Nations. [file 2049-9957-3-26-S1.pdf]

Translation of the abstract into the six official working languages of the United Nations

أثر التدخل المجتمعي في معرفة فيروس نقص المناعة البشرية والموقف منه وانتقاله

ريحانة أ سلام وسارة هارون وهاشم ه أحمد و جي ك داس وذو الفقار أ بوتة

ملخص:

يقدر عدد الناس الحاملين لفيروس نقص المناعة المكتسبة سنة 2012 ب35,3 مليون شخصا ، في حين يبلغ عدد المصابين الجدد بهذا الفيروس بمليونين تقريبا. يمكّن التدخل المجتمعي(CBIs) من أجل الوقاية من فيروس نقص المناعة ومراقبته من زيادة فرص الحصول على العناية الصحية وتسهيل توفيرها للأشخاص المعرضين للخطر أو المصابين به بالفعل. تقيّم هذه الوثيقة أثر التدخل المجتمعي في معرفة فيروس نقص المناعة البشرية والموقف منه وانتقاله. و لقد أدرجنا 39 دراسة عن الأنشطة التعليمية وجلسات الإرشاد والزيارات المنزلية والمراقبة والمجموعات النسائية وقيادة الأقران وأنشطة توعية الشارع في المجتمعات المحلية التي تهدف إلى زيادة الوعي بعوامل الخطر في انتقال فيروس نقص المناعة مع ضمان الامتثال للعلاج. وتشير نتائج المراجعة التي قمنا بها إلى نجاعة التدخل المجتمعي في زيادة الوعي بمرض نقص المناعة المكتسبة والحد من المخاطرفي تحسين المعارف والمواقف والممارسات والنتائج كما يتضح ذلك من زيادة متوسط درجات المعرفة لفيروس نقص المناعة البشرية(SMD:0.66, 95% IC: 0.25 , 1.07) ومن نسبة زيادة اللقاءات الجنسية المحميّة(RR :1.19 ومن نسبة الزيادة في استعمال الواقي الذكري (SMD:0.96 , 95% CI:1.13, 1.25) 95% IC: , 0.03, 1.58) وتراجع وتيرة الاتصال الجنسي(RR:1.19, 95% IC: 1.13, 1,25). كما أظهرت الدراسة أنه ليس للتدخل المجتمعي أي أثر هام على درجة الكفاءة الذاتية والتواصل حسب ما تم تقييمها من قبل دراسات فردية تضمنتها التحاليل. لقد وجدنا أدلة محدودة جدا عن إدارة مجتمعية للسكان المصابين بفيروس نقص المناعة المكتسبة والوقاية من انتقال المرض من الأم إلى الطفل(MTCT) للنساء الحوامل المصابين بفيروس نقص المناعة المكتسبة. يقترح الاستنتاج النوعي أن تأسيس لدعم مجتمعي في بداية برامج الوقاية من مرض نقص المناعة المكتسبة يؤدي إلى قبول المجتمع لها مع الالتزام بها. لقد تمت المناداة بضرورة وجود تربية وقائية من فيروس نقص المناعة المكتسبة في المدارس وبتوزيع وسائل منع الحمل كإستراتيجيات ممكنة لاستهداف المجموعات الشبابية ذات المخاطر العالية. إن التدخل المجتمعي لناجع في تحسين المعارف والمواقف ونتائج الممارسات. وتركز الدراسات المستقبلية على تقييم منصات توفرها المجتمعات المحلية من أجل الوقاية من انتقال العدوى من الأم إلى الطفل(MTCT) وعلى عدة نماذج صاعدة من الرعاية من أجل تحسين نتائج الاعتلال والوفيات.

Translated from English version into Arabic by malika2012, through

TRANSLATORS WITHOUT BORDERS

社区干预对 HIV 知识、态度和传播的影响

Rehana A Salam, Sarah Haroon, Hashim H Ahmed, Jai K Das, Zulfiqar A Bhutta

摘要

截至到 2012 年，全球约有 353 亿 HIV 感染者，其中约两百万为新发感染者，以社区为基础的预防和控制 HIV 干预措施（CBIs）可有效的减少 HIV 传播和流行，本研究拟评估 CBIs 对 HIV 知信行和传播流行的影响。共计 39 个研究被纳入，这些研究包括采用行为教育、检测咨询、家庭访问、行为监测、女性小组、同伴教育、街道外展服务等方法来提高人们对 HIV/AIDS 危险因素的认识，加强 HIV/AIDS 治疗的依从性。研究显示，通过 CBIs，增加人们对 HIV 的认识，降低 HIV 的传播风险，可有效的增加人们对 HIV/AIDS 的知信行，主要体现在以下几个方面：增加 HIV/AIDS 知识评分(SMD: 0.66, 95% CI: 0.25, 1.07)，增加保护性性行为(RR: 1.19, 95% CI: 1.13, 1.25)，增加避孕套的使用(SMD: 0.96, 95% CI: 0.03, 1.58)，降低性交频率(RR: 0.76, 95% CI: 0.61, 0.96)。分析显示，CBIs 对自我效能评分和个体沟通评估方面没有显著影响。以社区为基础的对 HIV 感染者的管理和预防控制 HIV 的母婴传播（MTCT）方面的研究报道较少。综合定性研究显示，在 HIV/AIDS 预防项目地区建立社区支持可以提高社区的参与和接受度。针对高危青年群体，以学校为基础的预防艾滋病教育和避孕宣传作为一个潜在的战略目标应当提倡。CBIs 在改善人们对 HIV/AIDS 知信行方面是有效的，进一步的研究应当关注评估以社区为平台的预防 HIV 的母婴传播的有效性以及各种新出现的增加发病率和死亡率的保健模式。

Translated from English version into Chinese by Tian Li-guang, through

CHINESE CENTER FOR DISEASE CONTROL AND PREVENTION
NATIONAL INSTITUTE FOR PARASITIC DISEASES

Impact des interventions basées communauté[1] sur la connaissance, les attitudes et la transmission du virus VIH

Rehana A Salam, Sarah Haroon, Hashim H Ahmed, Jai K Das, Zulfiqar A Bhutta

Résumé

En 2012, on estimait qu'il y avait 35,2 millions de personnes vivant avec le virus du VIH, avec environ deux millions de nouveaux cas d'infection signalés. Les interventions pour la prévention et le contrôle du virus du VIH basées communauté rendent meilleurs l'accès et la disponibilité des soins médicaux pour les populations à risque ou déjà infectées par le virus. Ce travail évalue l'impact des interventions basées communauté sur la connaissance, les attitudes, et la transmission du virus VIH. Nous avons inclus 39 études portant sur des activités éducatives, des séances-conseil, des visites à domicile, du mentoring, des associations féminines, des techniques de leadership par des pairs, et des activités de sensibilisation en milieux urbains par le biais de communautés, visant à éduquer sur les facteurs de risque HIV/AID et assurer une bonne adhésion au traitement. Nos conclusions indiquent que les interventions basées communauté permettent d'accroître la sensibilisation au VIH, contribuent à la réduction des risques, et sont efficaces pour améliorer les connaissances, les attitudes et les pratiques, comme en témoignent l'amélioration des scores de connaissance moyens pour le VIH/sida (DMS: 0,66, 95% IC : 0,25, 1,07), l'augmentation des échanges sexuels protégés (RR : 1,19, 95% IC : 1,13, 1,25), la meilleure utilisation des préservatifs (DMS : 0,96, 95% IC : 0,03, 1,58), et la diminution de la fréquence des rapports sexuels (RR : 0,76, 95% IC : 0,61, 0,96). L'analyse a montré que les interventions basées communauté n'avait aucun impact significatif sur les scores d'auto-discipline et de communication ainsi évalués dans les différentes études incluses dans l'analyse. Nous avons trouvé très peu de résultats en gestion axée communauté sur les populations infectées par le virus du VIH et sur la prévention de la transmission mère-enfant (TME) pour les femmes enceintes infectées par le virus du VIH. La synthèse qualitative nous indique que la mise en place d'un soutien communautaire en début de programme de prévention sur le virus VIH conduisait à un engagement et à l'acceptation de la part de la communauté. L'éducation en milieu scolaire sur la prévention contre le virus VIH et la distribution de contraceptifs ont

[1] Terme anglais (CBI) Community-Based Interventions

également été préconisés comme des stratégies potentielles pour cibler le groupe de jeunes à haut risque. Les interventions basées communauté sont efficaces pour améliorer la connaissance, les attitudes et les pratiques. Des études à venir devraient tenter d'évaluer l'efficacité des plateformes basées communauté de diffusion d'informations sur la transmission du virus d'une mère à son enfant, et des divers modèles émergents de soins pour améliorer les taux de morbidité et la mortalité.

Translated from English version into French by Corina Roe, through

TRANSLATORS
WITHOUT BORDERS

Влияние вмешательства общины на знания о ВИЧ, отношение к проблеме ВИЧ и передачу ВИЧ

Рехана А. Салам, Сара Хаурн, Хашим Х. Ахмед, Джай К. Дас, Зульфикар А. Бхутта

Аннотация

В 2012 г. примерно 35,3 миллионов человек являлись носителями ВИЧ, а также было выявлено примерно два миллиона новых случаев инфицирования ВИЧ. Вмешательство общины (ВО) для профилактики и контроля ВИЧ позволяет группам риска и лицам, уже инфицированным ВИЧ, получить более обширный доступ к медицинскому обслуживанию и большую доступность лечения. В данном исследовании оценивается влияние ВО на знания о ВИЧ, отношение к проблеме ВИЧ и передачу вируса. Мы включили сюда 39 исследований образовательных процессов, консультационных сеансов, посещений домов, руководства группами женщин, изучения лидерства среди групп сверстников, а также акций, направленных на жителей улицы, предпринятых усилиями общин, которые были нацелены на повышение информирования о факторах риска, касающихся ВИЧ/СПИДа, и обеспечение соблюдения медицинских рекомендаций. Итоги нашего обзора показали, что в работе по увеличению информированности о ВИЧ и снижении факторов риска ВО было эффективно в повышении знания, улучшении отношения к проблеме и практических результатов, как показали цифры об увеличении среднестатистического знания о ВИЧ/СПИДе (величина эффекта: 0,66, 95% ВО: 0,25, 1,07), увеличении количества защищенных сексуальных контактов (отношение рисков: 1,19, 95% ВО: 1,13, 1,25), увеличении случаев использования презервативов (величина эффекта: 0,96, 95% ВО: 0,03, 1,58) и снижения частоты половых актов (отношение рисков: 0,76, 95% ВО: 0,61, 0,96). Анализ показал, что ВО не имело никакого значительного эффекта на показатели самоэффективности и взаимодействия с окружающими, по оценкам отдельных исследований, включенных в анализ. Мы нашли очень мало свидетельства работы общины с ВИЧ-инфицированными людьми или профилактики передачи вируса от матери к ребенку (ПМР) для ВИЧ-инфицированных беременных женщин. Количественный синтез предполагает, что появление поддержки общины на заре программ профилактики ВИЧ ведет к принятию ВИЧ-инфицированных

общиной и вовлечению их в общественную жизнь. Образовательные программы по профилактике ВИЧ в школах и раздача контрацептивов также считаются потенциальными стратегиями, которые могут быть направлены на группу повышенного риска среди молодежи. ВО эффективно при повышении знаний, улучшения отношения к проблеме и практических результатов. Будущие исследования должны сосредоточиться на эффективности программных платформ общины для профилактики ПМР и различных развивающихся моделях лечения для улучшения показателей мобильности и снижения смертности.

Translated from English version into Russian by Mayya Shlyakhter, through

TRANSLATORS
WITHOUT BORDERS

El impacto de las intervenciones basadas en la comunidad sobre el conocimiento, las actitudes y la transmisión del VIH

Rehana A. Salam, Sarah Haroon, Hashim H. Ahmed, Jai K. Das, Zulfiqar A. Bhutta

Resumen

En 2012, se estimó que 35,3 millones de personas vivían con VIH y se detectaron aproximadamente 2 millones de nuevas infecciones causadas por este virus. Las intervenciones basadas en la comunidad (IBC) para la prevención y el control del VIH aumentan y mejoran el acceso a la atención médica de las poblaciones expuestas o infectadas con el virus. Este artículo analiza el impacto que las intervenciones basadas en la comunidad tienen sobre el conocimiento, las actitudes y la transmisión del VIH. Se incluyeron 39 estudios sobre actividades educativas, sesiones de asesoramiento, visitas domésticas, programas de tutorías, grupos de mujeres, liderazgo entre iguales y actividades de divulgación a pie de calle en contextos comunitarios con el objetivo de incrementar la concientización de la comunidad sobre los factores de riesgo del VIH/SIDA y garantizar la observancia del tratamiento. Los resultados sugieren que las IBC destinadas a aumentar la concientización sobre el VIH y reducir el riesgo de contagio son eficaces para mejorar el conocimiento, las actitudes y las prácticas, como lo demuestra el aumento del promedio de las puntuaciones sobre el conocimiento del VIH/SIDA (DME: 0.66, 95 % IC: 0.25-1.07), el aumento de las relaciones sexuales con protección (RR: 1.19, 95 % IC: 1.13-1.25), el aumento del uso de preservativos (DME: 0.96, 95 % IC: 0.03-1.58) y la disminución del número de encuentros sexuales (RR: 0.76, 95 % IC: 0.61-0.96). El análisis revela que las IBC no han tenido ningún impacto significativo en las puntuaciones sobre autoeficacia y comunicación según se observó en los estudios individuales que se incluyeron en el análisis. Se hallaron muy pocas pruebas sobre la gestión basada en la comunidad de las poblaciones infectadas con VIH y la prevención de la transmisión materno-infantil (PTMI) en el caso de las mujeres embarazadas infectadas con VIH. Los estudios sistemáticos cualitativos sugieren que la prestación de asistencia comunitaria desde el inicio de los programas de prevención del VIH favorece la aceptación y el compromiso de la comunidad. La impartición de programas de prevención del VIH a nivel escolar y la distribución de contraceptivos también se han identificado como posibles estrategias para llegar a la población joven de alto riesgo. Las IBC son efectivas para mejorar el conocimiento, las actitudes y las prácticas relacionadas con el VIH. Futuros estudios se centrarán en

la evaluación de la efectividad de las plataformas de prestaciones comunitarias para la PTMI y de varios modelos asistenciales emergentes para reducir las tasas de morbilidad y mortalidad.

Translated from English version into Spanish by Ma. Victoria Porro Rodríguez, through

TRANSLATORS
WITHOUT BORDERS
